# The Effective Treatment of Purpurin on Inflammation and Adjuvant-Induced Arthritis

**DOI:** 10.3390/molecules28010366

**Published:** 2023-01-02

**Authors:** Weiya Zeng, Caihong Shen, Suifen Mo, Chen Ni, Ying Lin, Yuan Fang, Huiling Yang, Guihua Luo, Luhua Xiao, Ruoting Zhan, Ping Yan

**Affiliations:** 1 College of Traditional Chinese Medicine, Guangzhou University of Chinese Medicine, Guangzhou 510006, China; 2 Key Laboratory of Chinese Medicinal Resources from Lingnan, Guangzhou University of Chinese Medicine, Ministry of Education, Guangzhou 510006, China; 3 Joint Laboratory of Nation Engineering Research Center for the Pharmaceutics of Traditional Chinese Medicines, Guangzhou 510006, China

**Keywords:** purpurin, rheumatoid arthritis, molecular docking, AIA rat

## Abstract

*Rubia cordifolia* L. (Rubiaceae), one of the traditional anti-rheumatic herbal medicines in China, has been used to treat rheumatoid arthritis (RA) since ancient times. Purpurin, an active compound of *Rubia cordifolia* L., has been identified in previous studies and exerts antibacterial, antigenotoxic, anticancer, and antioxidant effects. However, the efficacy and the underlying mechanism of purpurin to alleviate RA are unclear. In this study, the effect of purpurin on inflammation was investigated using macrophage RAW264.7 inflammatory cells, induced by lipopolysaccharide (LPS), and adjuvant-induced arthritis (AIA) rat was established to explore the effect of purpurin on joint damage and immune disorders; the network pharmacology and molecular docking were integrated to dig out the prospective target. Purpurin showed significantly anti-inflammatory effect by reducing the content of IL-6, TNF-α, and IL-1β and increasing IL-10. Besides, purpurin obviously improved joint injury and hypotoxicity in the liver and spleen and regulated the level of FOXP3 and CD4+/CD8+. Furthermore, purpurin reduced the MMP3 content of AIA rats. Network pharmacology and molecular docking also suggested that MMP3 may be the key target of purpurin against RA. The results of this study strongly indicated that purpurin has a potential effect on anti-RA.

## 1. Introduction

Rheumatoid arthritis (RA) is one of the chronic autoimmune and systemic inflammatory diseases [1]. RA characterized by inflammation, swelling, degeneration of cartilage and bone of joints, and weak tendons and ligaments [2], will lead to a reduced quality of life. Recent studies have reported that more than 1.5% of the population worldwide suffers from RA [3]. While it has been demonstrated that the key cause of RA is mainly related to genetic, environment, innate and adaptive immune response [4], metabolism [5], inflammation [6,7], oxidative stress [8], and hormone effect [9], the etiology and pathogenesis of this systemic disorder are still a major concern for researchers, and it is necessary to explore the mechanism of RA based on clinical data.

The medication to treat RA includes glucocorticoids (GCs) and nonsteroidal anti-inflammatory drugs (NSAIDs), which are mainly used to improve inflammation and pain. However, NSAIDs have various side effects and may affect the lung, liver, and spleen with long-term administration [10]. GCs are characterized by anti-inflammatory activity but also bring several adverse effects [11]. With the growing demand for drugs to treat RA, research interest in exploring the mechanisms of RA and discovering new drugs has increased.

**Figure 1:**
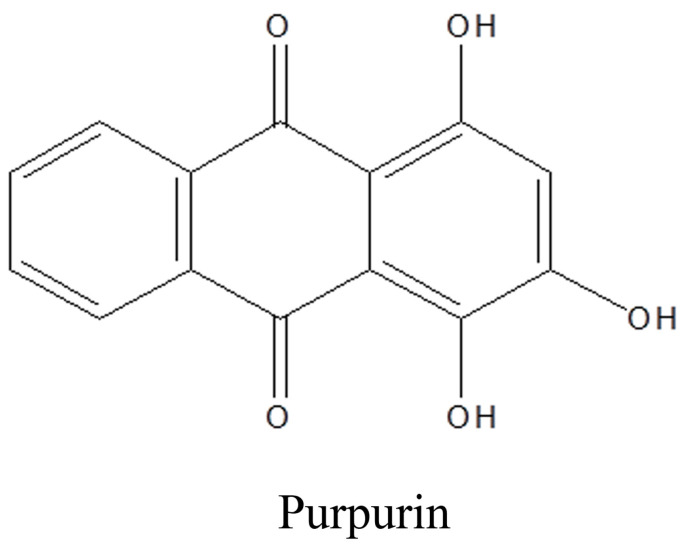
The chemical structure of purpurin.

The efficacy of traditional Chinese medicine (TCM) against chronic diseases, especially rheumatism, is evident [12]. More and more active compounds of TCM were applied in the clinic, such as rhein [13], triptolide [14], artemisinin [15], and berberin [16] etc. *Rubia cordifolia* L. (Rubiaceae) is a traditional anti-rheumatic herbal medicine in China, and its unique therapeutic effects have been confirmed by numerous studies [17]. In our previous study, the ethanol extract of *R. cordifolia* L. has a significant therapeutic effect on the adjuvant-induced arthritis (AIA) model of rats, and purpurin was identified as its active component [18,19]. Purpurin belongs to the class of organic compounds known as hydroxyanthraquinones (Figure 1). Scientific studies have reported that purpurin has obvious antibacterial, antigenotoxic, anticancer, and antioxidant effects [20,21,22]. Besides, purpurin suppressed atopic dermatitis by TNF-α/IFN-γ-induced inflammation in HaCaT cells via inhibition of the activation of protein kinase B (AKT), mitogen-activated protein kinase (MAPKs), and nuclear factor kappa-light-chain-enhancer of activated B (NF-κB) [23]. Emodin is a compound with a similar structure to purpurin, and has proved to be an effective treatment for RA by affecting inflammatory cytokines and T cells [24]. However, the mechanism of purpurin in relieving RA is still unknown.

Network pharmacology and molecular docking are powerful bioinformatics tools to discover all potential targets, functions and mechanisms of bioactive components for disease treatment [25,26]. In this study, LPS-induced inflammatory cells and AIA rat models were used to evaluate the effectiveness of purpurin on anti-RA, and network pharmacology and molecular docking were combined to explore the potential targets of purpurin against RA and provided a related basis theory for further studies.

## 2. Results

### 2.1. The Effect of Purpurin on LPS-Induced Inflammatory Cell Model

**Figure 2:**
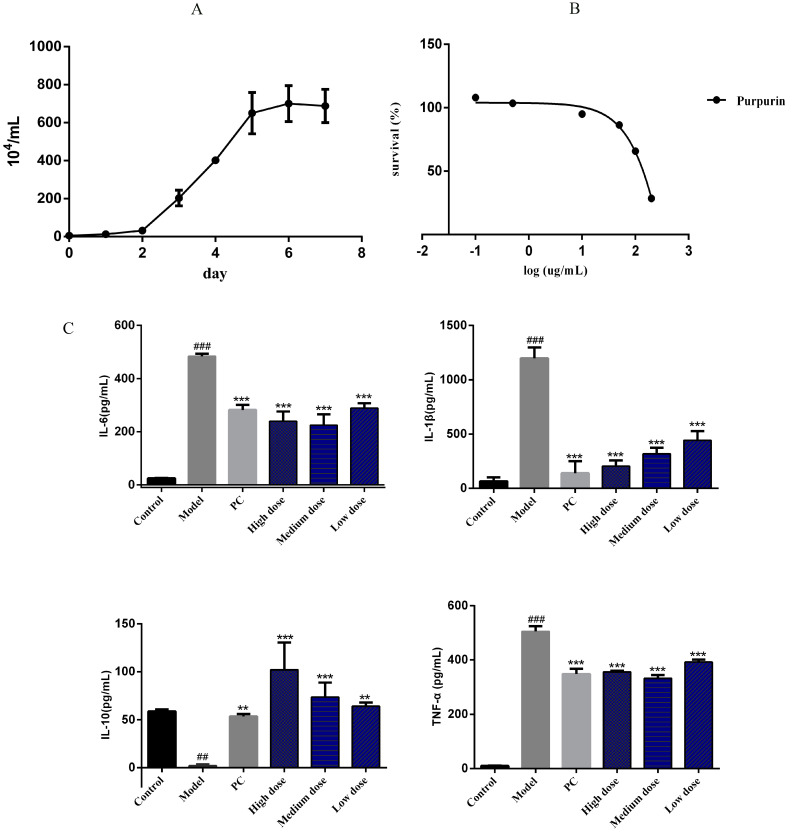
The effect of purpurin on inflammatory factors. (**A**) The growth curve of RAW 264.7 cells, *n* = 3. (**B**) Assessment of purpurin cytotoxicity using MTT assay. The tested concentrations of purpurin were 200, 100, 50, 10, 0.5, and 0.1 μg/mL, respectively, *n* = 3. (**C**) The anti-inflammatory effect of purpurin, the purpurin concentrations in the high-, medium-, and low-dose groups were 1, 0.5, and 0.1 μg/mL, respectively. Model: LPS group, PC: positive control group. Data are represented as mean ± SD. ^###^ *p* < 0.001, ^##^ *p* < 0.01 vs. control. *** *p* < 0.001, ** *p* < 0.01 vs. model.

The growth curve showed that the rapid growth period of cells appeared at the second day to the fifth day (Figure 2A), and the IC50 of purpurin was 131.9 μg/mL with a concentration range of 200, 100, 50, 10, 0.5, and 0.1 μg/mL (Figure 2B). In order to explore the anti-inflammatory effect of purpurin, interleukin (IL)-6, interleukin (IL-10), tumor necrosis factor (TNF)-α and interleukin (IL-1β) were measured in this study. When lipopolysaccharide (LPS) stimulated cells, the content of IL-6, TNF-α and IL-1β were significantly increased, while the content of IL-10 was decreased, but with the intervention of purpurin, the content of IL-6, TNF-α and IL-1β were obviously decreased and IL-10 was increased (Figure 2C).

### 2.2. Treatment of Purpurin on AIA Rats

**Figure 3:**
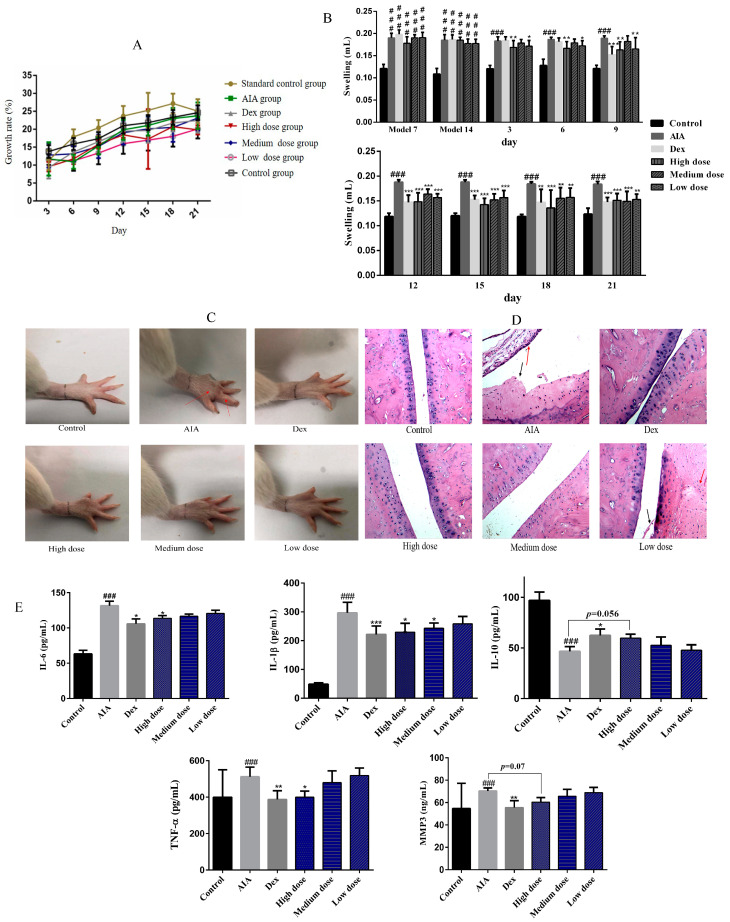
Effect of purpurin on weight, foot swelling, and joint damage in rats. (**A**) The changes in the weight of rats during administration. (**B**) The foot swelling of rats. (**C**) The appearance of foot in different groups after administration of the respective drugs for 21 days, paw swelling of AIA rat was marked by a red arrow. (**D**) H&E staining (magnification: 100-fold) of the joints after administration of the respective drugs for 21 days, red arrow: inflammatory cell infiltration, black arrow: cartilage destruction. (**E**) The effect of purpurin on IL-6, IL-1β, IL-10, TNF-α and MMP3 of AIA rats. Dex: dexamethasone; AIA: adjuvant-induced arthritis. Data are represented as mean ± SD. ^###^ *p* < 0.001 vs. control. *** *p* < 0.001, ** *p* < 0.01, * *p* < 0.05 vs. model, *n* = 6.

The results revealed that purpurin had no significant effect on the body weight of rats (Figure 3A). On the 7th and 14th days after CFA injection, AIA rats showed obvious paw swelling. Then, with the treatment of dexamethasone (Dex) and purpurin, the redness and swelling of rat paws were almost invisible (Figure 3B,C). Hematoxylin-eosin staining (HE) revealed intact joints with a smooth surface and a thin layer of hyaline cartilage in normal rats. However, under the influence of complete Freund’s adjuvant (CFA), inflammatory cell infiltration (red arrow) and cartilage destruction (black arrow) were observed in AIA rats. In the Dex group, there was some exudate in the inferior joint cavity, but the synovial cells on the cartilage surface without damage. With the treatment of purpurin, the damage of the joint surface of rats was gradually alleviated, and the synovial fibrosis was faded, and the inflammatory cells were almost invisible with the high- and medium-dose purpurin (Figure 3D). In addition, purpurin reduced the content of IL-6, IL-1β, TNF-α and matrix metallopeptidase 3 (MMP3) of AIA rats and increased the level of IL-10 (Figure 3E).

### 2.3. The Effect of Purpurin on Spleen and Liver of Rats

**Figure 4:**
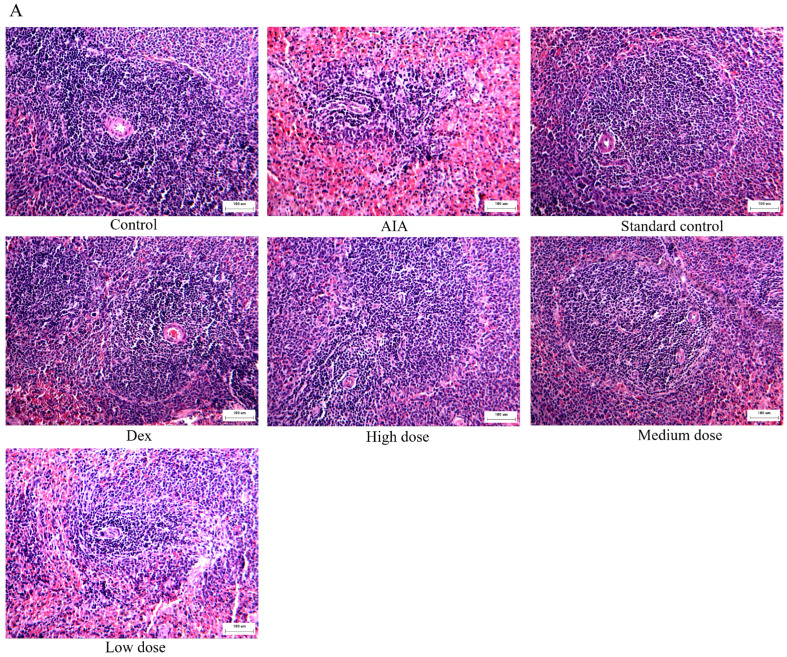

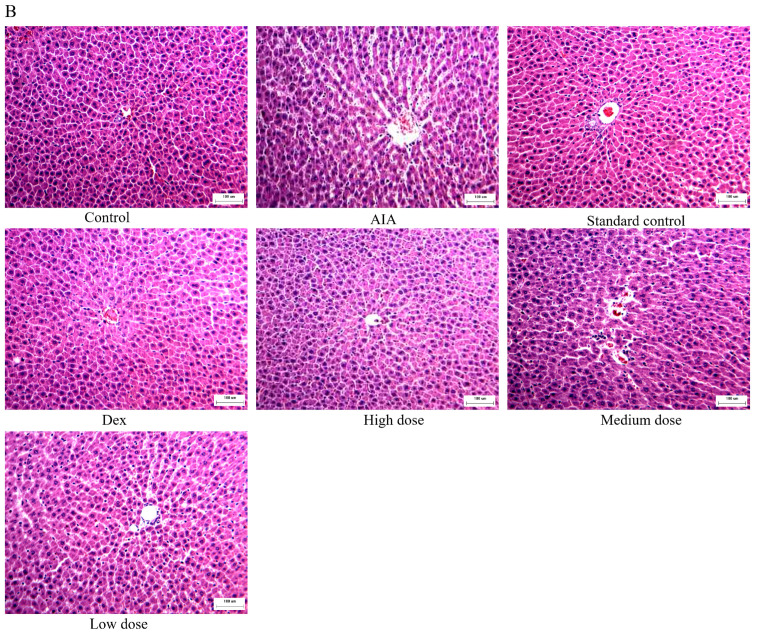
The influence on the liver and spleen after purpurin administration. (**A**) H&E staining of the spleen (magnification: 100-fold). (**B**) H&E staining of the liver (magnification: 100-fold). Dex: dexamethasone; AIA: adjuvant-induced arthritis; *n* = 6 in each group.

In order to evaluate the safety of purpurin, we analyzed its effects on the spleen and liver by HE staining. In the AIA group, the quantity of splenic corpuscles and lymph in spleen tissue were decreased, the germinal center in the splenic corpuscles was not obvious, and the splenic sinus in the red pulp expanded and congested of rats. However, the lesion of spleen tissue was significantly improved in the purpurin treatment groups. Furthermore, compared to the control group, the standard control group (administered with high dose purpurin in normal rats) did not show obvious damage in the spleen of rats (Figure 4A).

The liver structure and histology of rats were normal in the control group, and the liver plate, liver sinus and blood vessels were intact. However, with the influence of CFA, the liver of AIA rats performed atrophic hepatocytes with smaller volume, some inflammatory cell infiltration in the Disse’s space, and the structure of the hepatic plate was disordered. Compared to the AIA rats, purpurin gradually improved the structural and histological abnormalities of the liver, especially in the high-dose group. In the standard control group, the morphology of the liver of rats did not significantly change due to the high dose purpurin (Figure 4B).

### 2.4. The Immunomodulatory Effect of Purpurin

**Figure 5:**
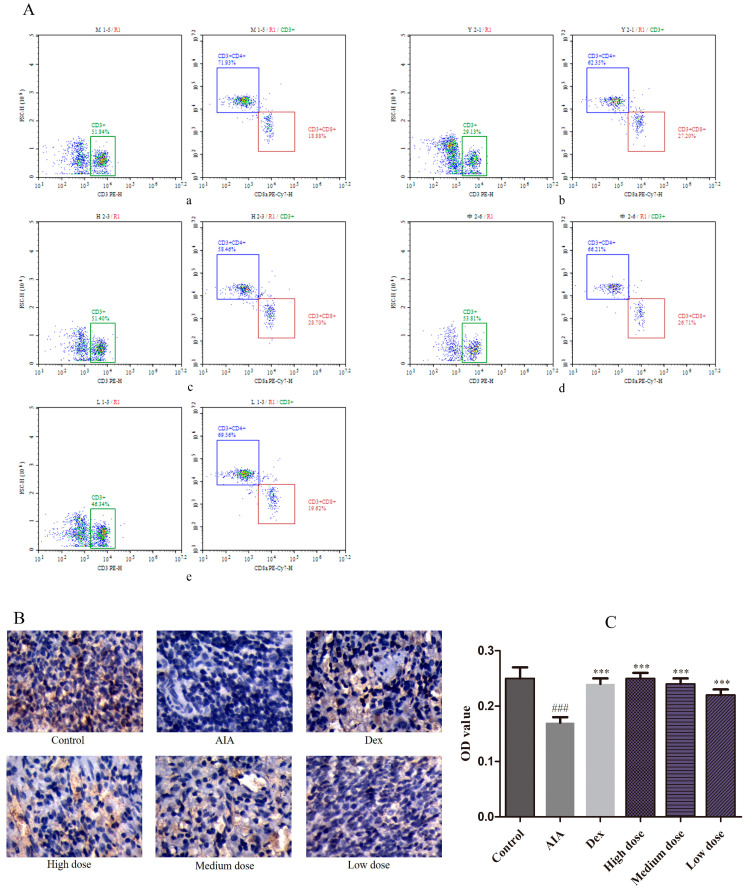
Effects of purpurin on CD4+ and CD8+ T cells, and FOXP3 contents. (**A**) The ratio of CD4+/CD8+ estimated using flow cytometry. (**a**) AIA group, (**b**) Dex group, (**c**) high-dose purpurin group, (**d**) medium-dose purpurin group, and (**e**) low-dose purpurin group. (**B**) Immunohistochemical staining of FOXP3 in the spleen tissue of rats in each group (magnification: 100-fold). (**C**) The optical density (OD) value of each group. Dex: dexamethasone; AIA: adjuvant-induced arthritis; Data are represented as mean ± SD. ^###^ *p* < 0.001 vs. control, *** *p* < 0.001 vs. model, *n* = 6 in each group.

To assess the immunomodulatory effect of purpurin, forkhead box protein 3 (FOXP3) and CD4+/CD8+ levels were estimated in this study. The CD4+/CD8+ was 3.81% in the AIA group. In the Dex group, the proportion of CD4+ was reduced and CD8+ was increased; the value of CD4+/CD8+ was decreased to 2.29%. Nevertheless, the abnormal expression was reversed with purpurin administration, the value of CD4+/CD8+ in high-, medium-, and low-dose purpurin groups was 2.04%, 2.48%, and 2.80%, respectively (Figure 5A, Table 1). FOXP3 in the spleen was stained with IHC in yellow–brown particles (Figure 5B) and displayed by OD value (Figure 5C). Compared to the control group, the FOXP3 expression of AIA group was significantly decreased. In contrast, the expression of FOXP3 in the Dex group and purpurin group increased significantly compared to the AIA group.

### 2.5. Identification and Analysis of Active RA Genes, Potential Targets and Key Target

**Figure 6:**
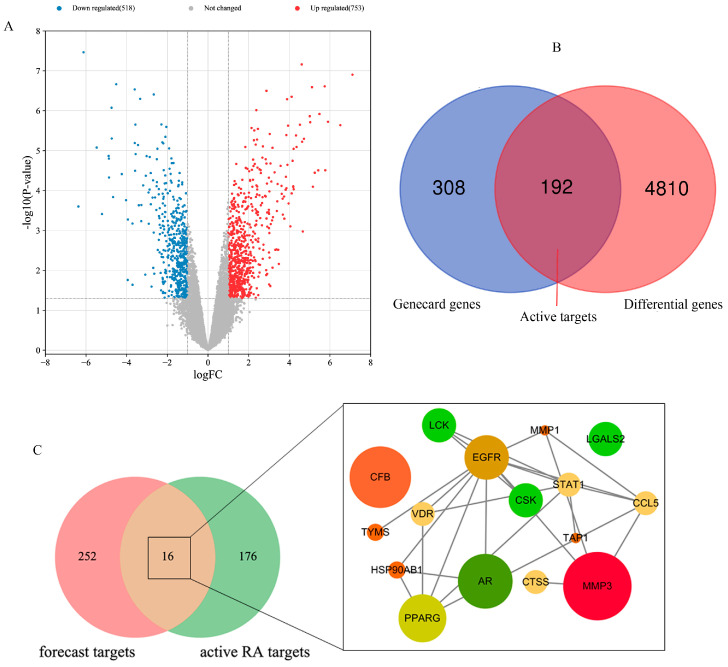
Analysis of RA genes and forecast targets of purpurin against RA, and functional characterization of potential targets of purpurin against RA. (**A**) Volcano-plot representation of differential gene expression. (**B**) Venn diagram depicting coincident genes of differential genes (top 500) and related genes from the GeneCard database. (**C**) Venn diagram depicting coincident genes of purpurin forecast targets and active RA genes.

**Figure 7:**
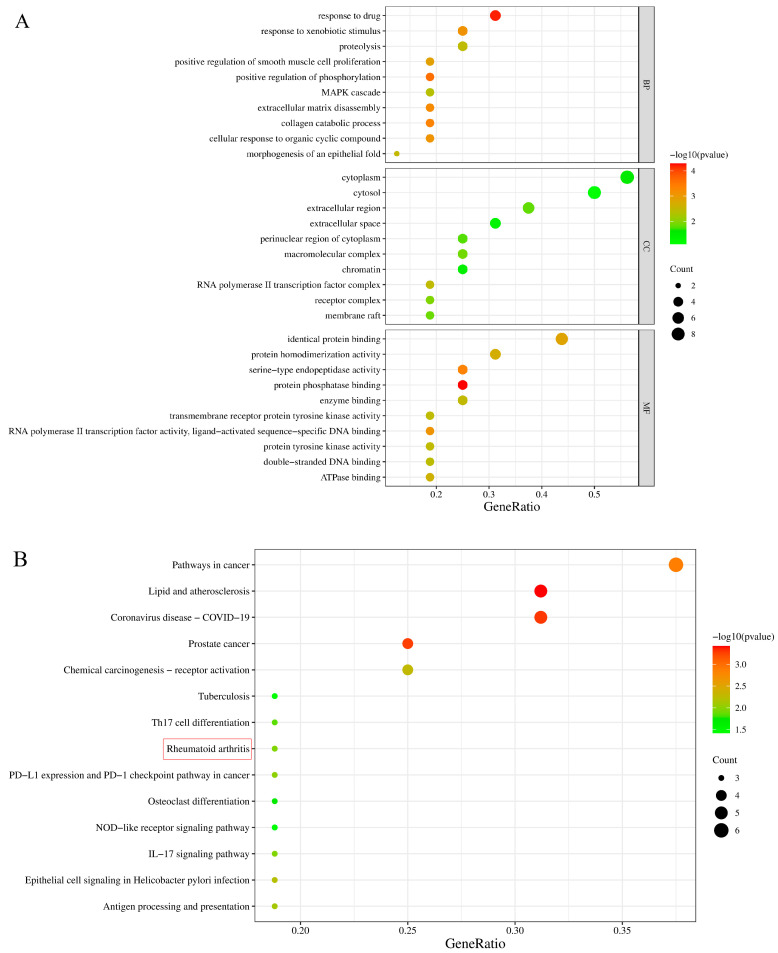
The functional characterization of potential targets of purpurin against RA. (**A**) Gene ontology analysis of potential targets of purpurin, biological process (BP), cell component (CC), molecular function (MF). (**B**) Kyoto Encyclopedia of Genes and Genomes (KEGG) pathway of potential genes of purpurin.

In order to research the potential and key target of purpurin against RA, relevant database analysis and network pharmacology were conducted. A total of 1271 differentially expressed genes were identified using the GEO database (Figure 6A), and 5002 related genes were obtained in Genecard database. 192 active RA genes were obtained by collecting the overlapping genes of GEO database and Genecard database (Figure 6B). The PharmMapper database provided 268 forecast targets of purpurin. By analyzing the forecast targets and active RA genes, 16 potential targets of purpurin against RA were acquired, including VDR, LGALS2, CCL5, CSK, CTSS, LCK, MMP3, CFB, EGFR, TYMS, TAP1, AR, HSP90AB1, PPARG, MMP1 and STAT1, of which MMP3 was considered as the key target as it has the highest fit score with purpurin (Figure 6C, Table S1). Potential targets were submitted to the David database for GO and KEGG enrichment analysis, purpurin affected a series of biological process, such as response to drug, positive regulation of phosphorylation, and collagen catabolic process (Figure 7A). Additionally, the KEGG pathway analysis showed 14 pathways (*p* < 0.05) related to potential targets (Figure 7B), and CCL5, MMP3 and MMP1 were enriched in the rheumatoid arthritis pathway (Figure S1).

### 2.6. Binding of Purpurin to Key Target

**Figure 8:**
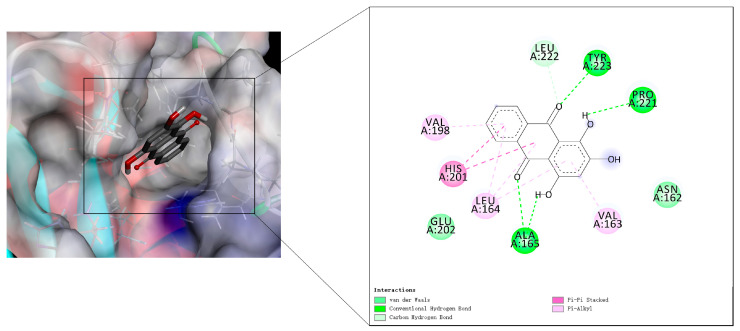
The interaction of purpurin and MMP3.

The molecular docking was conducted to uncover the under mechanism of purpurin on key target MMP3. The results showed that purpurin was in the pocket and interacted with amino acid residues of MMP3, purpurin formed hydrogen bond with the amino acid residue Ala165, Tyr223 and Pro221, formed carbon hydrogen bond with Leu222, formed two π-π bond with His201, and generated van der Waals interaction with Glu202 and Asn162, formed π-alkyl bond with Leu164, Val163 and Val198, and the binding energy between purpurin and MMP3 was −6.52 kcal/mol (Figure 8).

## 3. Discussion

Patients with RA exhibit redness, swelling, and pain in joints. The progress of RA involves various changes in pathology and physiology, and a wide range of cells participate in this progress, including monocytes/macrophages, FLS, T cells, and B cells, which produce several pro-inflammatory cytokines and other proteolytic enzymes [27]. Pro-inflammatory cytokines, especially TNF-α, IL-1β and IL-6, play an important role in the progression of RA patients [28]. The macrophages were activated by the immune complexes formed with anti-cyclic citrullinated peptide antibody and rheumatoid factor, then triggering the release of inflammatory cytokines [29]. The model of stimulating macrophages with LPS was mostly used to investigate inflammation in vitro [30,31]. TNF-α is considered as one of the key regulatory factors of RA pathogenesis, and participated in multiple bioprocess in RA development [32]. In RA patients, IL-6 can be continuously produced with TNF-α stimulation in synovial fibroblasts [33]. IL-1β was produced by macrophages and can stimulate macrophages in turn to aggravate inflammation, and result in cartilage damage by activating chondrocytes [34]. IL-10 possessed anti-inflammatory activity in RA pathogenesis, the level always increased when inflammation occurs [35]. In this study, purpurin showed considerable effects on TNF-α, IL-1β, IL-6 and IL-10; the great potential anti-inflammatory effects of purpurin are worth tapping into for further study.

AIA is a classic experimental polyarthritis model similar to RA, which has many pathological characteristics of RA, such as swelling of the limb, joint inflammation, synovial hyperplasia, and cartilage injury, and GCs had therapeutic effects in AIA rats [36,37,38]. Besides, CFA injection caused multiple joint and systemic inflammation, including lesions in the spleen and liver, GCs treatment altered liver transcription and ameliorated liver lesions in AIA rats [39]. In our study, purpurin alleviated the foot swelling and improved joint damage of AIA rats, and has little toxicity to spleen and liver, even improved the hepatocyte atrophy, inflammatory cell infiltration and liver plate structure disorder of liver tissue, and eased the abnormal of the splenic corpuscle, dilatation and widening of the splenic sinus in the red medullary, and congestion of spleen.

Additionally, RA is a kind of autoimmune disease, and the immune homeostasis and preventing autoimmunity were inseparable from Treg cells. CD4+ T cells regulate the inflammatory environment in RA through various subsets, CD4+/CD8+ is associated with immune dysfunction, an increased CD4+/CD8+ ratio has been implicated in patients with RA, while the development and suppressive activity of Treg cells require the master regulator FOXP3 [40,41,42]. In this study, FOXP3 expression was increased and the ratio of CD4+ and CD8+ was decreased by purpurin treatment, which further supported its potential efficacies of against RA.

Furthermore, MMP3 is known as stromelysin-1, and plays an important role in joint and bone injury, and radiological erosion, as well as a biomarker reflect RA disease; matrix metalloproteinase (MMPs) was secreted when synovial fibroblasts were activated by pro-inflammatory cytokines such as TNF and IL-6. The level of MMP3 usually up regulated in RA patients [43,44] (Table S1). Dehydroevodiamine is an effective compound of *Euodiae Fructus* and showed obvious reduction on the MMP3 of AIA rats [45]. Emodin was isolated from Radix et Rhizoma Rhei, and was similar to purpurin in structure, which possessed 9,10-dianthrone; this study showed that emodin significantly inhibited the expression of MMP3 [46]. It seems to be a bright prospect for compounds in medicine herb to reduce the content of MMP3. In addition, the study of MMP3 inhibitors has always been a concern for researchers. In inhibitor design studies, aryl rings and fewer substituents of compound for fit to MMP3 S1′ pocket, and aryl rings connected with Val198 and Leu164 in MMP3, flexible or semi flexible ring or long chain structure capable of hydrophobic interaction with various S1-S2′ residue side chains, and sulfonamide partially enhances the hydrogen bond with Leu164 [47,48]. The inhibitor can also interact with Asn162, Val163, Leu164, Ala165 and Leu222 of S2′ pocket hydrophobic layer [49]. The binding energy between MMP3 and co-crystallized ligand was −7.66 kcal/mol. In general, binding energies below −5.0 kcal/mol demonstrated that the 2 molecules are well connected [50]. According to the interaction study of co-crystallized ligand, the amino acid of MMP3 domain active site including Ala165, Leu222, Leu218, Leu164, Thr215 and Ala217, and phenyl ring of ligand was at the bottom of the S1′ pocket [51]. In this study, the level of MMP3 was reduced by purpurin, and molecular docking indicated that purpurin had a good connection with MMP3, the aryl ring of purpurin toward to the S1′ pocket in MMP3, and formed an π-alkyl bond with Val198 and Leu164, and on the other side, the aryl ring of purpurin also formed an π-alkyl bond with Val163 and Leu164, and generated van der Waals between purpurin and Asn162. In addition, purpurin also formed a hydrogen bond with Ala165, Pro221 and Tyr223 in MMP3. Therefore, the effect of purpurin on MMP3 and its mechanism could be evaluated and optimized by in vivo and in vitro experiments in the future.

## 4. Materials and Methods

### 4.1. Regents, Antibodies, and Drugs

Purpurin (purity > 98%) and carboxymethylcellulose sodium (CMC-Na) were purchased from Shanghai Winherb Medical Technology Co., Ltd. (Shanghai, China), and dexamethasone (Dex) was bought from Guangdong South China Pharmaceutical Co., Ltd. (Dongguan, China). Dulbecco’s modified eagle medium (DMEM), Fetal Bovine Serum (FBS), penicillin-streptomycin, and 3-(4,5-dimethylthiazol-2-yl)-2,5-diphenyltetrazolium bromide (MTT) were purchased from Gibco (New York, NY, USA). Indomethacin, complete Freund’s adjuvant (CFA, 1 mg/mL of heat-inactivated Mycobacterium tuberculosis dissolved in 85% paraffin oil and 15% mannide monooleate), lipopolysaccharide (LPS), and dimethyl sulfoxide (DMSO) were obtained from Sigma-Aldrich Chemical Company (St. Louis, MO, USA). The antibodies of CD3 PE, CD4 FITC, and CD8 PE-CY7 for flow cytometry were purchased from BioLegend (California, CA, USA). Bicinchoninic Acid Kit for Protein Determination (BCA kit) was bought from CST (Boston, MA, USA). The anti-FOXP3 antibody was purchased from BioLegend; ultra streptavidin HRP detection kit (rabbit, DAB), IL-6, IL-10, TNF-α, and IL-1β enzyme-linked immunosorbent assay (ELISA) kits for cell experiment were obtained from R&D Systems (Minneapolis, MN, USA), the IL-6, IL-10, TNF-α, MMP3 and IL-1β ELISA kits for animal experiment were purchased from Jingmei Biotechnology Co., Ltd. (Jiangsu, China); and red blood cell lysate buffer was bought from Solarbio Science and Technology Co., Ltd. (Beijing, China), 10% ethylene diamine tetraacetic acid (EDTA) solution was purchased from.

### 4.2. Cell Culture

The macrophage RAW 264.7 line (Cell bank of the Chinese Academy of Sciences, Shanghai, China) was cultured in 89% DMEM, 10% FBS, and 1% penicillin-streptomycin and kept at 37 °C and in a humidified atmosphere of 5% CO_2_ [52]. For the cell growth assay, 1 × 10^3^ cells/well suspension were seeded in 96 well plates, and the cell numbers were measured every day and continuously for 7 days. For inflammatory factors assay, 1 × 10^6^ cells/well were incubated for 24 h in 6-well plates, 0.1 μg/mL LPS and drug were added for 18 h. For cytotoxicity assay, we took cells from the logarithmic growth stage and inoculated them (5 × 10^3^ cells/well) in 96-well plates for 24 h, then purpurin with different concentrations (200, 100, 50, 10, 0.5, and 0.1 μg/mL) was added in triplicate and incubated for 18 h, the medium was replaced by 200 μL medium containing 0.5 mg/mL MTT solution and incubated for 4 h, afterward, the supernatant was removed, and 100 μL DMSO was added, then measured in 540 nm wavelength as quickly as possible [53]. The purpurin stock solution was dissolved in DMSO (the final concentration of DMSO was under 0.1%), and the working solution was diluted in the medium.

### 4.3. ELISA

For cell experiment, the cell culture medium supernatant was collected after 18 h of purpurin administration for inflammatory factors detection. For the animal experiment, the blood of rats was centrifuged at 1500× *g* for 10 min at 4 °C, and the supernatant was collected for the detection of TNF-α, IL-6, IL-10, IL-1β and MMP3. Levels of TNF-α, IL-6, IL-10, IL-1β and MMP3 were detected using respective ELISA kits following the manufacturer’s instructions.

### 4.4. Experimental Animals

Adult male Wistar rats weighing 170–200 g were purchased from the Southern Medical University Experimental Animal Center (Guangzhou, China). The certificate number was SCXK (Yue) 2016-0041. The animal experiments were conducted under experimental conditions of the experimental animal center of Guangzhou University of Chinese Medicine (Guangzhou, China) and approved by the Experimental Animal Ethics Committee of the Guangzhou University of Chinese Medicine (authorization number 20191112005). The rats were provided sufficient ordinary feed and water every day. The temperature was maintained at 20–25 °C, the relative humidity was 40–60%, and the experiment was carried out after seven days of adaptive feeding. After adaptive feeding, the right hind paw of rats was injected with 0.1 mL CFA to established AIA model [19,54,55,56], while the control rats were injected with an equivalent volume of saline.

The AIA rats were randomly divided into five groups (*n* = 8 per group) including te AIA group, AIA + Dex group (1.25 mg/kg), AIA + purpurin high-dose group (80 mg/kg), AIA + purpurin medium-dose group (60 mg/kg), and AIA + purpurin low-dose group (40 mg/kg), the normal rats were separated to two groups (*n* = 8 per group), control group and standard control group (80 mg/kg purpurin); the standard control group was established to observe the toxicity of purpurin for normal rats. Purpurin powder was dissolved to 4 mg/mL, 6 mg/mL and 8 mg/mL with 0.5% CMC-Na solution. After CFA injection for 14 days, rats were given corresponding drugs by gavage for 21 days. The weight and paw swelling of rats were recorded every 3 days. Paw swelling was measured by the volumetric method. The weight gain rate was calculated with the following formula: Growth rate (%) = (Mt − M0)/M0, where M0 and Mt are weight on the day of RCE administration (day 0) and on day t after administration, respectively.

### 4.5. Histopathological Examination (HE) and Immunohistochemistry (IHC)

After administration for 21 days, the liver and spleen tissues and joints of rats were submitted to fixation with 4% paraformaldehyde. The joints of rats were decalcified using 10% EDTA solution. For HE of the liver, spleen, and joint, the tissues were dehydrated with different concentrations of alcohol, permeabilized with xylene, embedded in paraffin, stained with hematoxylin and eosin, and sealed [19]. The IHC assay was used to detect FOXP3 expression in the spleen. The tissues were heated, fixed, deparaffinized, rehydrated, and blocked with 3% goat serum after antigen retrieval and incubated with primary antibodies overnight at 4 °C. Afterward, the secondary antibodies were added and incubated at room temperature. Then the BCA working solution was used to stain the tissue. Sections were counterstained with hematoxylin. The expression of FOXP3 was detected using IPP 6.0 image analysis software and presented using optical density (OD) value [57].

### 4.6. Flow Cytometric Analysis

The peripheral blood of rats was collected by heparin sodium anticoagulant tube. Approximately 50 μL of peripheral blood of rats was placed in the flow tube, and CD3 PE, CD4 FITC, and CD8 PE-CY7 antibodies (3 μL each) were added to the tube, respectively. The tubes were then incubated at 4 °C in the dark for 30 min, and subsequently, 1x red blood cell lysate was added and maintained for 15 min.

### 4.7. Identification of Genes, Enrichment Analyzes and Identifying Key Target of Purpurin against RA

The genes of RA were obtained from GEO dataset (GSE1919, https://www.ncbi.nlm.nih.gov/geo/, accessed on 12 November 2022) and Genecard database (https://www.genecards.org/, accessed on 15 November 2022). The differential genes of RA were screened by the ‘limma’ package of R-language Bioconductor with false discovery rate < 0.05 and | logfold change (FC) | > 1 [58]. In order to conform the RA–associated genes, the active RA genes of this study were collected by the coincident genes of the GEO dataset (top 500) and Genecard database. The forecast target of purpurin was acquired from PharmMapper platform (http://lilab-ecust.cn/pharmmapper/index.html, accessed on 15 November 2022). The coincident targets of forecast targets and active RA genes were potential targets of purpurin against RA. The gene ontology (GO) biological process and KEGG pathways of potential targets were analyzed using DAVID function database (https://david.ncifcrf.gov/, accessed on 15 November 2022). The protein-protein interaction network (PPI) of potential targets was obtained by String (https://cn.string-db.org/, accessed on 15 November 2022), then the network of potential targets and key target were analyzed using Cytoscape 3.5.1 software [59].

### 4.8. Molecular Docking

The MMP3 protein structure (PDB ID: 2D1O) was obtained from the PDB database (https://www.rcsb.org/, accessed on 16 November 2022), and the purpurin was drawn by ChemBioDraw Ultra 14.0 (CambridgeSoft Corporation, Cambridge, MA, USA) and transformed into a 3D structure using ChemBio3d ultra14. 0 software. The structure of MMP3 was input to Discovery Studio 4.5 software (BIOVIA) for extracting the original ligand, and saved as pdb files respectively. The structure of protein and ligand were processed and saved as pdpqt file before docking by AutodockTools 1.5.6 (Scripps Research Institute) [60]. The binding site was set according to the co-crystallized ligand, the parameter of center grid box was x: 29.194, y: 7.769, z: 14.716, and with the box size of x: 50, y: 50, z: 50. Autodock 4.0 was used for docking, the remaining parameters were default when software was running, while the Discovery Studio 4.5 software was used to draw the optimal docking conformation and showed the molecular interaction.

### 4.9. Statistical Analysis

Data were analyzed by SPSS statistics 19.0 software and plotted using GraphPad Prism 6.0 software. One-way analysis of variance (ANOVA) used to calculate the significant difference among the groups. A *p*-value of less than 0.05 was considered significant. The data were expressed as mean ± standard deviations (SD).

## 5. Conclusions

The treatment dose of purpurin was not only less toxic to the growth, spleen, and liver of rats, but could also reduce foot swelling and improve joint injury of AIA rats. Purpurin played an anti-inflammatory role by inhibiting IL-6, TNF-α, and IL-1β and increasing IL-10, and regulated immune disorder by decreasing the CD4+/CD8+ ratio and increasing the FOXP3 level. Besides, purpurin could reduce the level of MMP3, and the network pharmacology and molecular docking also indicated the MMP3 could be a key target for further studies. Collectively, our findings suggested that purpurin can serve as a potential drug for the treatment of RA.

## Figures and Tables

**Table 1:** The ratio of CD4+/CD8+ of rats.

Group	Dose (mg/kg)	CD4+/CD8+
AIA	-	3.81
Dex	0.125	2.29
High dose	80	2.04
Medium dose	60	2.48
Low dose	40	2.80

Dex: dexamethasone; AIA: adjuvant-induced arthritis.

## Data Availability

Not applicable.

## Supplementary Materials

The following supporting information can be downloaded at: https://www.mdpi.com/article/10.3390/molecules28010366/s1, Table S1: The related parameters of potential targets. Figure S1: The related targets of purpurin in rheumatoid arthritis pathway. Supplementary method regarding the basis of dose usage in animal experiment.

## Sample Availability

Samples of the purpurin are available from the authors.
