# Supplementary material for: The Effective Treatment of Purpurin on Inflammation and Adjuvant-Induced Arthritis
Source: Molecules. 2023 Jan 2;28(1):366. doi: 10.3390/molecules28010366 (PMC9824476; doi:10.3390/molecules28010366)
Supplement: Supplementary file 1 [file molecules-28-00366-s001.zip › molecules-2081142-supplementary.pdf]

Supplementary material

Table S1. The related parameters of potential targets.

Uniprot ID	Gene	Fit score	logFC	P.Value
P11473	VDR	0.3706	1.2766293	0.000867
P05162	LGALS2	0.4659	4.3575678	0.00000809
P13501	CCL5	0.3718	4.1135285	0.000000446
P41240	CSK	0.4204	1.1829742	0.00012
P25774	CTSS	0.3899	1.4419218	0.00118
P06239	LCK	0.4769	2.7040002	0.00000783
P08254	MMP3	0.9937	4.3298607	0.00000415
P00751	CFB	0.9367	1.4727805	0.000227
P00533	EGFR	0.532	-1.5462788	0.00124
P04818	TYMS	0.309	1.8361032	0.0000081
Q03518	TAP1	0.2439	1.0969987	0.000687
P10275	AR	0.6422	-2.4185364	0.00172
P08238	HSP90AB1	0.2983	-0.7821443	0.00173
P37231	PPARG	0.5635	-1.5561872	0.000587
P03956	MMP1	0.2558	4.6654086	0.00106
P42224	STAT1	0.3489	1.9806674	0.000248

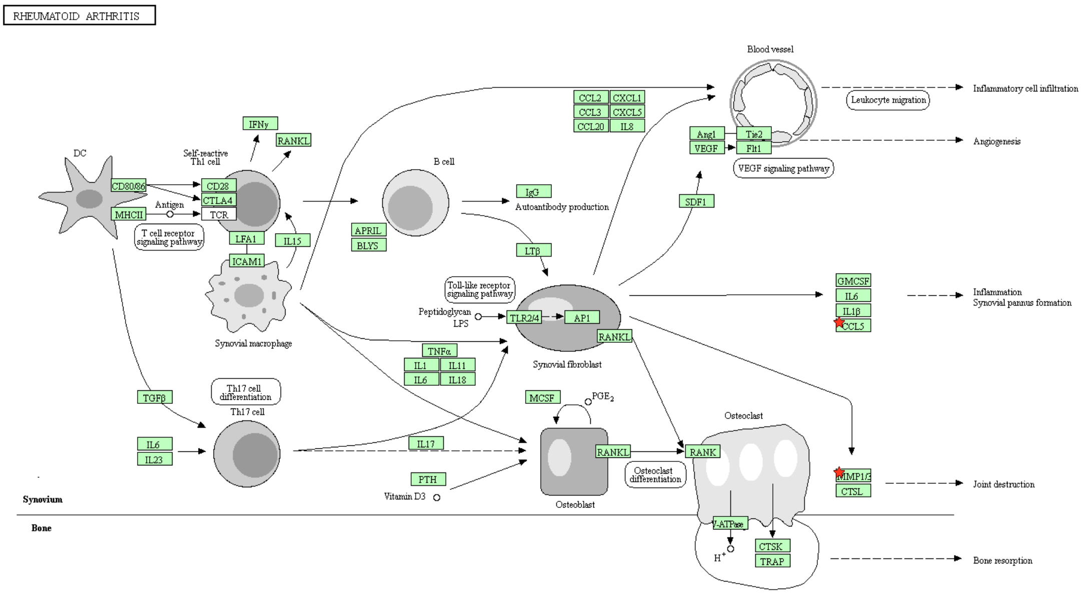

Figure S1. The related targets of purpurin in rheumatoid arthritis pathway. Analyzed in David database (https://david.ncifcrf.gov/).

Supplementary method:

The dose of purpurin for aniaml were based on the IC50 in cell experiment. According to the formula of (EC50×Bioavailability) / (mrat×8%) = IC50, the IC50 value of purpurin was 131.9 μg/ml, and the bioavailability of purpurin is 18.41%, which was obtained from TCMSP database (https://old.tcmsp-e.com/molecule.php?qn=6157), (EC50×18.41%)/(200 g×8%) = 131.9 μg/ml, EC50=11.463 mg/ml, the dose was 11.463 mg/ml/200 g=57.315 mg/kg, thus the dose of 40 mg/kg, 60 mg/kg and 80 mg/kg were applied in experiment.
